# Reconfigurable Meta-Coupler Employing Hybrid Metal-Graphene Metasurfaces

**DOI:** 10.1038/s41598-020-63660-x

**Published:** 2020-05-06

**Authors:** Mohammad Reza Tavakol, Amin Khavasi

**Affiliations:** Electrical Engineering Department, Sharif University of Technology, Tehran, 11155-4363 Iran

**Keywords:** Optoelectronic devices and components, Nanophotonics and plasmonics, Optics and photonics

## Abstract

Efficient excitation of surface wave (SW) remains one of the most challenging considerations in the photonics and plasmonics areas. Inspired by recent investigations of metasurfaces, we propose a hybrid metal-graphene transmitarray converting incident propagating wave (PW) to SW, as a solution for SW excitations–a meta-coupler. The structure comprises ultra-thin four-layer transparent metasurfaces in which H-shaped etched metal films together with graphene patches are employed, and also all four layers are identical. Full-wave simulations demonstrate that the suggested meta-coupler possesses an efficiency of 46% and a directivity of 19 dB, which is promising in the terahertz (THz) range. At the same time, in light of unique graphene characteristics, the proposed device is tunable and easily reconfigurable, i.e., the direction of converted SWs can be electrically switched from right to left and vice versa. We believe that this system responds to emerging applications such as THz communications and sensing, and furthermore the employed architecture introduce electrostatically tunable building blocks being able to develop graphene plasmonic components effectively.

## Introduction

Excitation and manipulation of surface waves (SWs), including surface plasmon polaritons (SPPs) and spoof SPPs which are the low-frequency counterparts of SPPs, have attracted ample interests in photonics and Terahertz (THz) scopes^1,2^. Two extraordinary and unique features of SPPs, subwavelength confinement and local field enhancement, have opened up next-generation applications, such as super-resolution imaging, biosensing, and integrated plasmonic circuits^3–5^. Although the behavior of metals does not lead to those extraordinary properties by themselves at lower frequencies (from GHz to mid-infrared range), some certain structured metals may provide them in light of spoof SPPs^2^. Therefore, same applications can be feasible at lower frequencies. As concerns SW generation developments, both efficient and unidirectional SW excitation is demanding and indeed challenging^6,7^, which originates from an incident propagating wave (PW). The main reason for this is the momentum mismatch between incident PWs and SW modes. Prism and grating couplers have conventionally resolved this issue forbidding direct coupling between PWs and SWs. Challenges in achieving an efficient and unidirectional coupling, nevertheless, have remained in these schemes. To the best of recent researches^7^, metasurface-based SW couplers, i.e., meta-couplers with their novel functionality would overcome these challenges in PW-SW conversion^8^.

To now, researches on metasurfaces, planar artificial surfaces modulating electromagnetic (EM) boundary conditions, have been growing owing to their promising functionalities^9–11^. Metasurface elements are usually subwavelength resonators that enable control over EM waves behavior through an specific boundary. These elements, so-called meta-atoms, can locally manipulate phase, amplitude, and polarization. Graphene, a monolayer of carbon atoms arranged in a honeycomb lattice^12^, has been incorporated recently into metasurfaces^13–15^ to make them more promising, operative and applicable in different scopes due to its fascinating optoelectronic properties, e.g., tunability and ability to support plasmons from mid-infrared to THz frequencies^16^. Consequently, tunable and reconfigurable devices and components with diverse capabilities can be realized^17–22^, one category of which is meta-couplers^23^. One class of graphene-based devices is featured by the metal-graphene hybridization where the EM coupling between metal structures and graphene is tailored^24^. This configuration provides various applications, where light-matter interactions can be controlled, e.g., ultrafast and spatial light modulation, electromagnetically induced transparency, and beam steering for THz and mid-infrared ranges^25–28^.

In this work, we propose a novel system for realizing a meta-coupler at THz range (*f * = 3 THz), which comprises four layers of an ultra-thin metasurface. Not only does our meta-coupler overwhelm the issues related to both efficiency and unidirectionality, but it also is reconfigurable, i.e., we can select the direction of the coupled SW. The reconfigurability–and tunability–of the meta-coupler is achieved by virtue of graphene patches participation in our design process. In this regard, our device can switch between two states corresponding two opposite directions for the SW propagations along the target waveguide. In our system, a dielectric-coated metal sheet–grounded dielectric slab– has been chosen as the target waveguide supporting SWs, and geometrically identical meta-atoms have constituted the meta-coupler. Based on the full-wave simulations, the proposed device behaves satisfactorily in terms of coupling efficiency (46%) as well as directivity (19 dB).

## Results and Discussions

**Figure 1:**
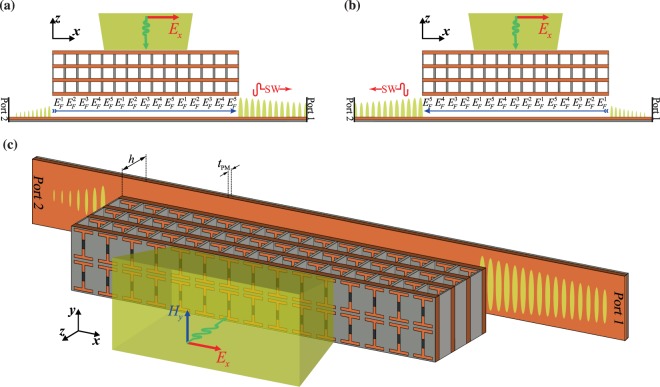
Working principle and configuration of the graphene-based meta-coupler for (**a**) state I and (**b**) state II. In states I and II, the incident propagating wave in the opposite of $z$-direction is coupled to ports 1 and 2, respectively. The Fermi level values of graphene patches in meta-atoms are labeled below them in each supercell. Orders of the Fermi level values corresponding to states I and II are in reverse to each other (the blue arrows in (a) and (b) indicate these orders). By changing the order of the Fermi level values from left-to-right to right-to-left, the device can be switched from state I to state II. (**c**) 3D schematic representation of the meta-coupler operating in state I. In these illustrations, the yellow regions depict illuminating $x$-polarized Gaussian beams.

Let us start considering the configuration schematically illustrated in Fig. 1. To provide appropriate phase gradient, a transmitarray–multiple stacked metasurfaces–is employed^29,30^, which grants us more degrees of freedom for miscellaneous functionalities^31–33^. We organized our proposed structure based on the array architecture, thanks to a given full control of the output beam phase provided by graphene patches incorporating with etched metal sheets. The transmitarray is composed of four similar layers spaced equally along the *z*-axis. Each layer has a contribution to form the expected transmitted beam phase profile to couple the input PW to SW at the target port/direction. Our proposed meta-coupler is able to select the output port and handle the input power to output power carried by an SW in the corresponding direction. We define states I and II which determine the functionality of the meta-coupler. Our meta-coupler is operating in state I when the incident beam couples to the guided SW at port 1, see Fig. 1(a). On the contrary, when the input beam couples to the guided SW at port 2, the meta-coupler is operating in state II, see Fig. 1(b). The graphene patches provide this reconfigurable functionality of the proposed meta-coupler. The meta-coupler configuration is geometrically uniform, i.e., geometric dimensions of elements in each meta-atom are identical through the full structure which supercells make up, and thus just the graphene Fermi level tuning can tailor the meta-atoms’ responses in order to implement the desired phase gradient profile. In this manner, the sequential order of Fermi level values indicated by blue arrows specifies the meta-coupler operating state, as depicted in Fig. 1(a,b). The meta-coupler positioned at an optimized distance of h above the target waveguide supporting SWs. Additionally, for the configuration illustrated in Fig. 1, the unwanted coupling to ports which are not our target is schematically displayed–in states I and II, the unwanted coupling occurs in ports 2 and 1, respectively, as exposed in Fig. 1(a,b). This undesired coupling degrades the directionality performance of the designed meta-coupler, and we are supposed to suppress it.

### Graphene-based meta-coupler design

A meta-coupler in which PW-SW coupling arises can be regarded as an extreme example of beam steering. Here, we interpret the PW-SW coupling process in light of the generalized Snell’s law of refraction for anomalous bending^9,34^:

1 $$\sin ({\theta }_{t})-\,\sin ({\theta }_{i})=\frac{1}{{k}_{0}}\frac{d\Phi }{dx}$$

where ${\theta }_{i}$ and ${\theta }_{t}$ are angles of incident and refraction, ${k}_{0}$ is the free space wavevector, and $d\Phi /dx$ is the phase gradient in the lateral direction. In the above equation, the phase gradient term provides a jump between the incident and transmitted parallel wavevectors, which leads to anomalous deflection. Clearly, we have a propagating transmitted wave unless the wavevector parallel component for that (${k}_{tx}={k}_{0}\,\sin \,{\theta }_{t}$) is larger than ${k}_{0}$, and in this apparent case, ${\theta }_{t}$ is real. Now consider the case in which phase gradient term is chosen in such a way that $|{k}_{tx}| > {k}_{0}$. As to this case, $|\sin \,{\theta }_{t}|\mathrm{ > 1}$, and therefore ${\theta }_{t}$ can be modeled by a complex angle, i.e., ${\theta }_{t}\mathrm{=90}+j{\alpha }_{t}$^35^. This condition corresponds to the SW induction process in which the phase gradient term compensates the momentum mismatch between incident PW and induced SW. This induced SW whose parallel wavevector is formed by a constant phase gradient ($\xi =d\Phi /dx$) can be coupled to a guided-wave structure supporting a bound state, e.g., a plasmonic surface or a corrugated metallic surface, when the condition $\beta ={k}_{{\rm{SW}}}={k}_{0}\,\sin \,{\theta }_{i}+\xi$ is satisfied–$\beta$ is the propagation constant for a bound state. Configurations that can perform such a PW-SW conversion are recently designed and studied for microwave and visible ranges, but fewer works developed for THz range^36^.

It is worth explaining how PW-SW conversion efficiency can be maximized. Firstly, we should strive to suppress impedance mismatch between the incidence medium and the device. To ensure that the unwanted reflection from the input port which degrades the meta-coupler performance, is minimized. Secondly, the lateral inhomogeneity of meta-coupler has to be introduced in a manner that minimizes momentum mismatch between the induced SW and the target waveguide eigenmode. Also, a careful implementation of the lateral inhomogeneity results in a better directional performance. Taking the mentioned rules into consideration, we should choose an optimized distance between the meta-coupler and the target system, which is denoted by $h$ in Fig. 1.

#### Unit cell

**Figure 2:**
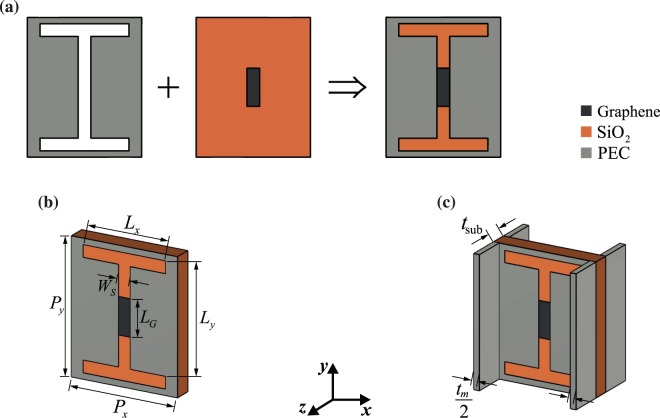
(**a**) Pictorial representation of how the proposed single-layer metasurface unit cell would be composed: to form the unit cell configuration, the complementary H-shape metal (PEC) is placed on the dielectric substrate on which graphene patches have been deposited. (**b**) Schematic of the proposed single-layer metasurface unit cell with geometric parameters ${P}_{x}=18$, ${P}_{y}=30$, ${L}_{x}=14$, ${L}_{y}=28$, ${W}_{S}=2$ and ${L}_{G}=6$, all in the unit of $\mu {\rm{m}}$. (**c**) Modified unit cell structure by adopting thin PEC separation walls to considerably decrease the coupling between adjacent cells. The dielectric substrate thickness, ${t}_{{\rm{sub}}}$, and metallic wall thickness, ${t}_{m}$ are 3 and 2 $\mu {\rm{m}}$, respectively.

As previously stated, to enhance meta-coupler efficiency, we have to reduce the impedance mismatch between the device and free space (the incident medium) as much as possible. Transparent windows and metasurfaces would be an unquestionable choice, which leads us to this goal^37,38^. Stacking metasurfaces whose transparencies are adequate, provides not only an acceptable amplitude but also full control of the phase at the output^29^. To this end, a transparent graphene-based metasurface is proposed here. The unit cell of our structure consists of a complementary H-shaped metal film and a graphene patch on a thin dielectric substrate made of SiO_2_ as schematically shown in Fig. 2(a). It should be noted that such hybrid configurations are realizable and practical. Figure 2(b) illustrates the 3D view of the unit cell in which the dimensions and details are depicted. As a minimal modification, thin metallic (silver) walls separating adjacent unit cells in the *x*-direction are also adopted to diminish inter-couplings between them. Meta-atoms would have a better performance due to this small modification which imposes a negligible variation in the unit cell response, see Fig. 2(c).

**Figure 3:**
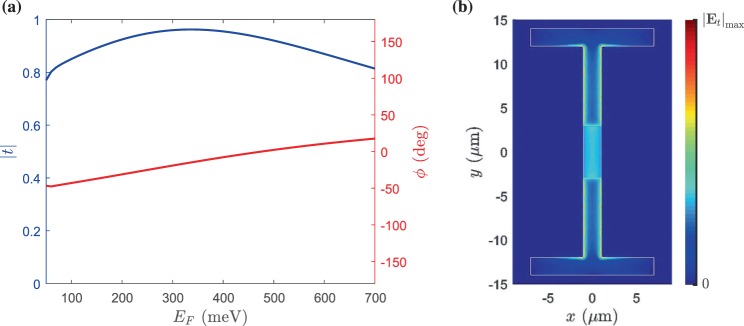
(**a**) Amplitude (blue line, left axis) and phase (red line, right axis) of the transmitted wave from the single-layer metasurface whose schematic is illustrated in Fig. 2 as a function of Fermi level of the graphene patch at $f=3$ THz. (**b**) The amplitude of the tangential electric field on the upper face of the proposed single-layer metasurface for the case in which the maximum transmission occurred. White lines specify the H-shape carved PEC region and graphene patch boundaries.

At low THz frequencies, the conductivity of metals is high enough to approximately model them as PECs^39^, and, on the other hand, the graphene shows plasmonic effects. In this way, the hybrid metal-graphene configurations in which the boundaries of metal and graphene touch each other may be exploited to develop emergent THz components, e.g., meta-couplers. Concerning our proposed unit cell structure, the electromagnetic interactions between the graphene patch and the H-shaped etched metal films play the main role in the transmission response. Consequently, the transmission response of the proposed single-layer unit cell can be adjusted by tuning the Fermi level of graphene patches, and this outcome can be seen from Fig. 3(a). Additionally, in Fig. 3(b), we show how the etched metal film together with the graphene patch form the tangential electric field distribution. The mean value of transmission amplitude over the 50–700 meV Fermi level range for the graphene patch is 0.89 (−1 dB), which is sufficient; however, the transmission phase shift is about 70°, which is too small for controlling the phase at the output. Consequently, by creating a transmitarray in light of this unit cell, a careful design may point us to a transmission response which grants us a phase control range of 360° with acceptable amplitude.

**Figure 4:**
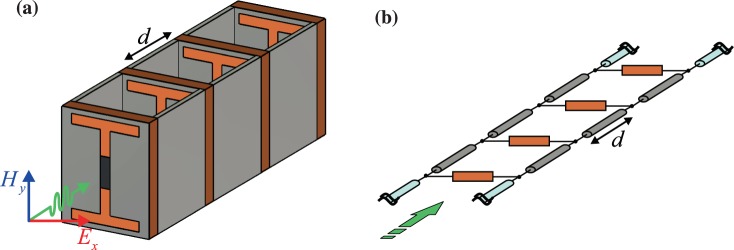
(**a**) The unit cell of the proposed transmitarray, which is composed of four layers of the metasurface whose unit cell is illustrated in Fig. 2. (**b**) The equivalent circuit model of the transmitarray configuration upon which we designed the device. In the transmitarray, layers (metasurfaces) are spaced to each other with equal distances (*d* = 17 μm).

Now, the single-layer graphene-based metasurface upon which we compose the transmitarray is specified. We extract the shunt admittance of this single-layer metasurface from full-wave simulations, and then make use of it in our design which is based on the circuit theory. Using transmission line models and that obtained admittance, we developed a four-layer metasurface whose layers are equally spaced with respect to the normal direction–*z*-axis direction. Figure 4(a,b) depict the unit cell of the transmitarray and its equivalent circuit model. To have a 360° phase control and adequate amplitude in the output, we choose *d* = 17 μm for the distance between every two adjacent layers as in our design procedure.

**Figure 5:**
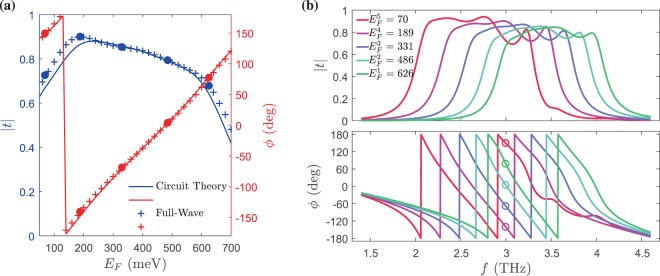
(**a**) Transmission amplitude (left axis) and phase (right axis) which is carried out based on the circuit model presented in Fig. 4(b) and full-wave simulations versus Fermi level of graphene patches for the proposed transmitarray at $f=3$ THz. The filled circles depict the selected Fermi level values, which should be set for meta-atoms. For the pluses, the transmission amplitude and phase values are computed using full-wave simulations. (**b**) Full-wave simulated transmission amplitude and phase responses of the structure whose unit cell is presented in Fig. 4(b) for five different graphene Fermi level values versus the frequency.

As plotted in Fig. 5(a), the transmission amplitude and phase response of the designed multi-layer unit cell behave desirably. The results carried out based on the transmission line model are in very good agreement with those obtained by full-wave simulations. For the unit cell, we select five Fermi level values with the same phase difference as 626, 486, 331, 189 and 70 meV, which are depicted by filled circles. Not only do these selected values cover 360° in the phase response, but they also have acceptable amplitudes. The average transmission amplitude for them is 0.8, which is larger than 0.7 (−3 dB).

Additionally, we plot the amplitude and phase of the transmission response as a function of frequency. As manifest in Fig. 5(b), the transparent window response shifts in frequency owing to the Fermi level changing of graphene patches in our transmitarray. Furthermore, it is evident from Fig. 5(b), that the variously shifted transparent windows correspond to five different values of Fermi level–626, 486, 331, 189 and 70 meV. The Fermi level values should be selected so that we have equally-spaced phase responses for a specific frequency point/range where all of the transparent windows corresponding to them permit. Herein, the phase change step between these five transparent windows is $\varDelta {\phi }=\mathrm{360/5}={72}^{\circ }$.

#### Meta-coupler

It is time to comprise our meta-coupler based on those picked Fermi level values according to their responses explained previously. The supercell of our geometrically uniform configuration comprises five meta-atoms for which the phase change step is chosen ${72}^{\circ }$. To meet the phase-matching condition, the supercell length (period) in the $x$-direction should be equal to the guided SW wavelength. Given the fact that the meta-atoms width is ${P}_{x}=18\,\mu {\rm{m}}$, the supercell length is $L=5{P}_{x}=90\,\mu {\rm{m}}$. At $f=3\,{\rm{THz}}$, simple calculations confirm that the meta-coupler phase gradient $\xi =2\pi /L$ is matched to the wavenumber of the bound state supported by the target waveguide, $\beta =1.11{k}_{0}$. Therefore the meta-coupler converts a normal incident PW (${\theta }_{i}=0$, so ${k}_{ix}=0$) into the SW. It should be noted that, for the target waveguide–the dielectric-coated metal sheet–functioning as the artificial plasmonic metal^40^, the dielectric is made of SiO_2_, the thickness of which is ${t}_{{\rm{PM}}}=8.2\,\mu {\rm{m}}$.

Throughout our device development, we keep the geometric features of our meta-coupler elements identical through the whole structure to, firstly, have a more straightforward design procedure, and secondly, make our device easily reconfigurable by the Fermi level of graphene patches, although this consideration limits our choices. Hence, the meta-coupler can provide either positive or negative phase gradient, and finally, we would be able to control the guiding direction of the coupled SW, which may be either in the $x$- or opposite of $x$-direction.

**Figure 6:**
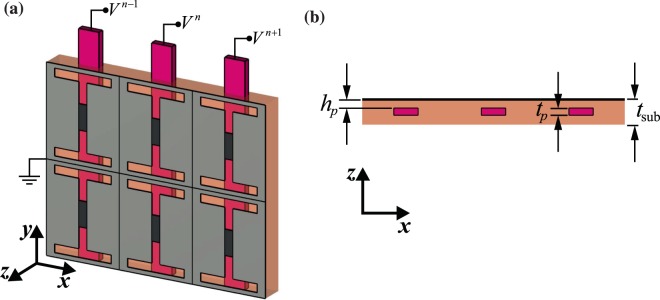
(**a**) Three-dimensional schematic of the biasing architecture in which ultrathin polysilicon pads are employed to apply DC voltages for each layer of a meta-atom. (**b**) Cross-section of a single layer in the meta-atom. The polysilicon pads whose thickness is ${t}_{p}\approx 100$ nm are indicated with red-violet color, and ${h}_{p}=50$ nm is the distance between them and the graphene patches.

In this regard, an architecture is developed to control the Fermi level of graphene patches electrically. The employed architecture is shown in Fig. 6. In each layer, ultra-thin polysilicon pads whose thickness is ${t}_{p}\approx 100$ nm are mounted in the ${{\rm{SiO}}}_{2}$ substrate in the vicinity of graphene patches. The graphene patches which are in direct contact with the carved metal films are grounded, and the DC voltages are applied to the polysilicon pads to control the Fermi levels electrostatically in meta-atoms. In each supercell, by applying proper DC bias voltages, the desired graphene Fermi level values in meta-atoms are arranged. The sequential order of bias voltages in each supercell specifies the state of the meta-coupler. The meta-coupler state would be switched when the direction of applied bias voltages is mirrored across the $x$-axis. Mirrored bias voltages lead to mirrored Fermi level values for meta-atoms, and finally, the generated SW direction is reversed as already indicated in Fig. 1(a,b). It is worth remarking that the transmission amplitude and phase, which are plotted in Fig. 5, are obtained for the unit cell in which the polysilicon pads are neglected. Nonetheless, the transmission coefficient, *t*, for the unit cell of the biasing structure where polysilicon pads are considered is almost the same as the results in Fig. 5 (see Supplementary Section 3).

**Figure 7:**
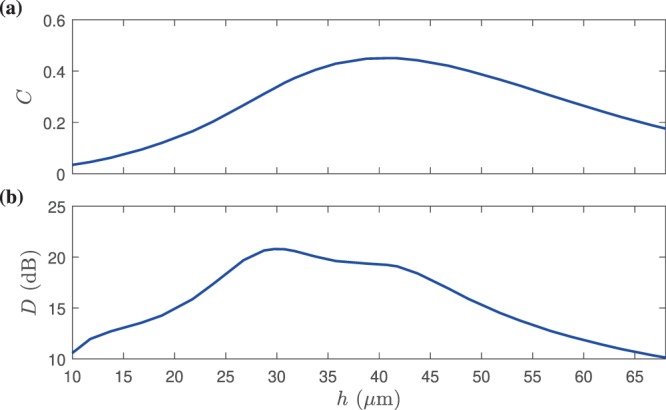
(**a**) Total coupling efficiency and (**b**) directivity of the meta-coupler composed of five supercells as a function of the gap distance of $h$ between the meta-coupler and target waveguide when $f=3$ THz and the incident Gaussian beam width, ${w}_{0}$ is 220 $\mu {\rm{m}}$. The three-dimensional view of such a configuration is comprehensible from Fig. 3(a), which operates in state I.

As the final step in our design procedure, we find the optimum gap distance $h$ between the meta-coupler and the target waveguide as previously mentioned. To optimize $h$, we should first define the two most influential parameters for this component, coupling efficiency $C$ and directivity $D$. Coupling efficiency $C$ is defined as the ratio between the power carried by the generated SW at the target port (herein, port 1 for state I) and that carried by the impinging PW. The directivity $D$ describing the directionality performance is also another important parameter, which defined as the ratio between the SW power flowing to the target port and the other port–directivity may be stated in dB scale as $10log(D)$. Ideally, the main objective is to maximize total coupling efficiency and directivity simultaneously with respect to $h$. To clarify how the gap distance $h$ between the device and target waveguide affects the performance, in Fig. 7(a,b), both coupling efficiency and directivity are plotted versus $h$ for finding the optimized distance. As can be observed, $h=40$ and $30\,\mu {\rm{m}}$ result in maximum efficiency and directivity, respectively. for $h=40\,\mu {\rm{m}}$, $D=19\,{\rm{dB}}$ and $C=\mathrm{46 \% }$, and also for $h=30\,\mu {\rm{m}}$, $D=21\,{\rm{dB}}$ and $C=\mathrm{36 \% }$. The optimum values for efficiency and directivity have not taken place at a single-point. However, we select $h=40\,\mu {\rm{m}}$ to maximize the coupling efficiency yet having a reasonable directivity ($D=19\,{\rm{dB}}$).

### Graphene-based meta-coupler performance

Designing and analyzing transmitarray, we compose the whole configuration where the meta-coupler performance investigated by means of full-wave simulations. Regarding that the meta-coupler in states I and II are the mirrored version of each other, it is required to investigate one of them, and thus the other state has the same performance. In this regard, we consider the meta-coupler operating in state I, whose 3D schematic as well as functionality were illustrated in Fig. 1(c) before. The device configuration that we want to study is made up of five supercells that each of them itself consists of five meta-atoms arranged sequentially in the $x$-direction. The meta-atoms opening up 360° phase control are previously determined (see Fig. 5(a)), whose difference is only the Fermi level values of graphene patches. Accordingly, an induced driven SW can be provided by a appropriate phase gradient profile matched to the propagation constant of our target waveguide ($\beta ={k}_{{\rm{SW}}}=1.11{k}_{0}$) merely by electrostatic biasing of graphene patches.

**Figure 8:**
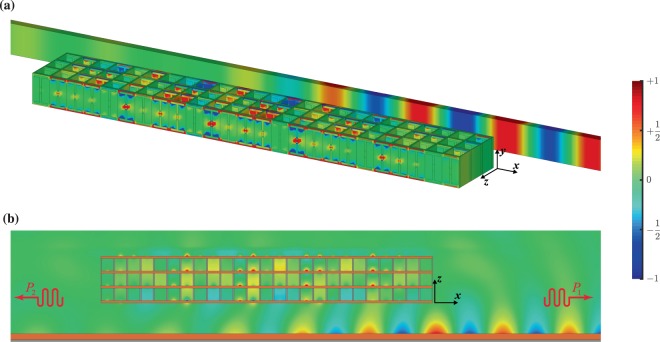
2D and 3D snapshots of the $y$-component of normalized magnetic field distribution, ${H}_{y}$ for the designed and optimized meta-coupler when the x-polarized Gaussian beam illuminates the structure in the opposite of $z$-direction. (**a**) Magnetic field profile on the structure sources and the target waveguide. (**b**) Magnetic field profile on the $xz$-plane. The meta-coupler is operating in state I.

The proposed structure is normally illuminated by an *x*-polarized Gaussian beam with beam waist ${w}_{0}=220\,\mu {\rm{m}}$ as the input PW, which is highlighted by yellow regions in Fig. 1(a–c). For the whole configuration, the *y*-component of the magnetic field pattern obtained by full-wave simulation at a working frequency of 3 THz is depicted in Fig. 8. In Fig. 8(b), the power carried by the coupled SWs in the $x$- and the opposite of $x$-direction are labeled by ${P}_{1}$ and ${P}_{2}$, respectively, for the proposed meta-coupler operating in state I. In Fig. 8(a,b), field distributions qualitatively bring to light that ${P}_{2}$ is negligible compared to ${P}_{1}$. In other words, the field patterns on lateral sides of the device graphically demonstrate that this meta-coupler has an outstanding directional behavior.

**Figure 9:**
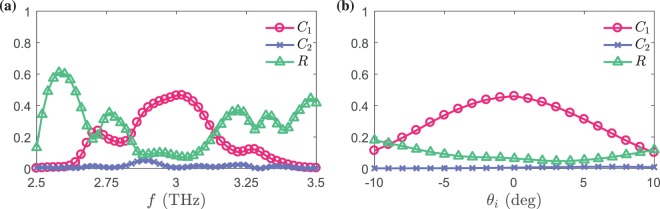
(**a**) The power reflection coefficient ($R$) and coupling efficiencies to port 1 and 2 (${C}_{1}$ and ${C}_{2}$) versus frequency for the meta-coupler operating in state 1, which is under normal illumination. (**b**) Those coefficients for the meta-coupler operating in state I as a function of the incident angle at $f=3$ THz.

Figure 9(a,b) demonstrates the robustness of the proposed device versus frequency and the incident angle ${\theta }_{i}$, where the reflected power, coupled power to the desired and undesired directions (${C}_{1}$ and ${C}_{2}$) are plotted. Again, these plots confirm the remarkable meta-coupler directivity, but this time with respect to both $f$ and ${\theta }_{i}$.

As an endpoint in the investigation of our meta-coupler performance, the achieved efficiency is larger than many recently proposed PW-SW converters or grating couplers^41–45^. Nevertheless, higher coupling efficiency have been reported in microwave or optical regimes^8,46^. But, it should be noted that our THz device is reconfigurable and tunable and it has reasonably high efficiency.

## Conclusion

In summary, as a starting point, we studied a hybrid graphene-metal metasurface as transparent windows upon which an efficient and reconfigurable meta-coupler is achievable. A transmitarray was developed by cascading four identical layers of those transparent metasurfaces in light of circuit theory to cover ∼360° transmission phase with an adequate amplitude. As a consequence, we designed a meta-coupler which solves PW-SW conversion issues, unwanted reflection and decoupling effects. The meta-coupler’s meta-atoms having electrical tunability granted by graphene patches in its structure is the cornerstone of our arrangement, where the intra-coupling between the graphene and the H-shaped etched metal film play the main role. Our proposed meta-coupler would convert the normally illuminated PW to the SW in the desired direction with efficiency of 46% and directivity of 19 dB just by electrostatic biasing of five graphene patches in each supercell. The versatility of the hybrid graphene-metal unit cell reveals a broad range of emerging applications in tunable, real-time, and integrated THz/optoelectronic systems such as more functional meta-couplers enabling polarization and wavelength splitting/controlling or even modulation (by nonlinear optics in the visible range).

## Methods

The proposed configurations are numerically calculated using the commercial package CST Microwave Studio. For unit cell structures simulations, to consider the inter-element coupling, we apply the unit cell boundaries as periodic boundary conditions along the *x*- and *y*- directions and Floquet ports in the *z*-direction. The unit cells transmission responses are obtained when these structures are normally illuminated by an *x*-polarized plane wave.

For the multi-layer unit cell analysis based on circuit theory, we firstly obtain the admittance matrix of the single-layer metasurface from the scattering parameters which are extracted based on full-wave simulations, as^47^:

2 $$[Y]=\frac{1}{{\eta }_{0}}([U]-[S]){([U]+[S])}^{-1}$$

where [*S*] is the scattering matrix, [*U*] is the unit, or identity, matrix, and η_0_ is the intrinsic impedance of vacuum. Considering the admittance matrix for the single-layer metasurface, then, we are able to calculate the transmission coefficient of the transmitarray unit cell in light of transmission line theory, whose circuit model was depicted in Fig. 4(b) before.

As to the full structure simulation, in order to terminate the whole domain, the begining and the end of the target waveguide in the *x*-direction are terminated using ports, and moreover, the periodic boundary condition as well as perfectly matched layers (PMLs), are applied in the $y$- and $z$-directions, respectively. The excitation is implemented by a Gaussian beam with a beam waist of ${w}_{0}=220\,\mu {\rm{m}}$, as a field source impinging the whole configuration. It should be pointed out that the efficiency and directivity of our proposed component are obtained by power field integration at the field source and ports faces.

The dielectric substrate used in our structures is made of ${{\rm{SiO}}}_{2}$ with relative permittivity ${\varepsilon }_{r}=3.9$ and thickness ${t}_{{\rm{sub}}}=3\,\mu {\rm{m}}$. Concerning modeling of polysilicon pads in the biasing architecture illustrated in Fig. 6, it is apropos mentioning that the embedded ones in ${{\rm{SiO}}}_{2}$ substrates would be reliably neglected due to being extremely thin and similar permittivity to that of ${{\rm{SiO}}}_{2}$ substrate^22,48^.

The carved thin metal films placed on each layer of the meta-coupler are modeled as (2D) PEC boundaries since metals, e.g., silver, nearly behave as PECs in the low THz region^39^.

The graphene is modeled as a conductive sheet with surface conductivity ${\sigma }_{g}$ by which tangential magnetic field boundary condition is modified. Consequently, in our simulation setups, we modeled graphene patches with surface impedances whose impedance values were determined by reversing their surface conductivities, i.e., $Z\mathrm{=1/}{\sigma }_{g}$. Based on the Kubo formula^49^, the conductivity is given by:

3 $${\sigma }_{g}=\frac{2{e}^{2}{k}_{B}T}{\pi {\hslash }^{2}}\frac{j}{j{\tau }^{-1}-\omega }\,\mathrm{ln}\left[2\,\cos \,h\left(\frac{{E}_{F}}{2{k}_{B}T}\right)\right]-\frac{j{e}^{2}}{4\pi \hslash }\,\mathrm{ln}\left[\frac{2{E}_{F}-\hslash (\omega -j{\tau }^{-1})}{2{E}_{F}+\hslash (\omega -j{\tau }^{-1})}\right]$$

where $e$ is the electron charge, ${k}_{B}$ is the Boltzmann constant, $T$ is the temperature, $\hslash$ is the reduced Planck constant, $\tau$ is the relaxation time, $\omega$ is the angular frequency and ${E}_{F}$ is the Fermi level. We assumed that the relaxation time $\tau =1\,{\rm{ps}}$ throughout this paper.

## Supplementray information

Supplementray information.
