# Supplementary material for: Reconfigurable Meta-Coupler Employing Hybrid Metal-Graphene Metasurfaces
Source: Sci Rep. 2020 May 6;10:7684. doi: 10.1038/s41598-020-63660-x (PMC7203159; doi:10.1038/s41598-020-63660-x)
Supplement: Supplementary file 1 — Supplementray information. [file 41598_2020_63660_MOESM1_ESM.pdf]

Supplementary Information

Reconfigurable Meta-Coupler Employing Hybrid Metal-Graphene Metasurfaces

Mohammad Reza Tavakol[1] and Amin Khavasi[1,*]
[1]*Electrical Engineering Department, Sharif University of Technology, Tehran, 11155-4363, Iran*
[*]*Corresponding author: khavasi@sharif.edu*

1. The Single-Layer Metasurface Development

1.1. The Unit Cell Design

We describe the design procedure in more details. First off, given the propagation constant of the SW in the target waveguide, the supercell length is determined, and $P_x = 18\ \mu\mathrm{m}$ is set then–here, we consider that the supercell length is equal to $5 \times P_x$. Secondly, the other dimension of the unit cell, P_y, has to be chosen. In this regard, we assume that $P_y = L_y + 2$ (in the unit of μm). Now, we study the unit cell response for the different values of L_x and L_y when the

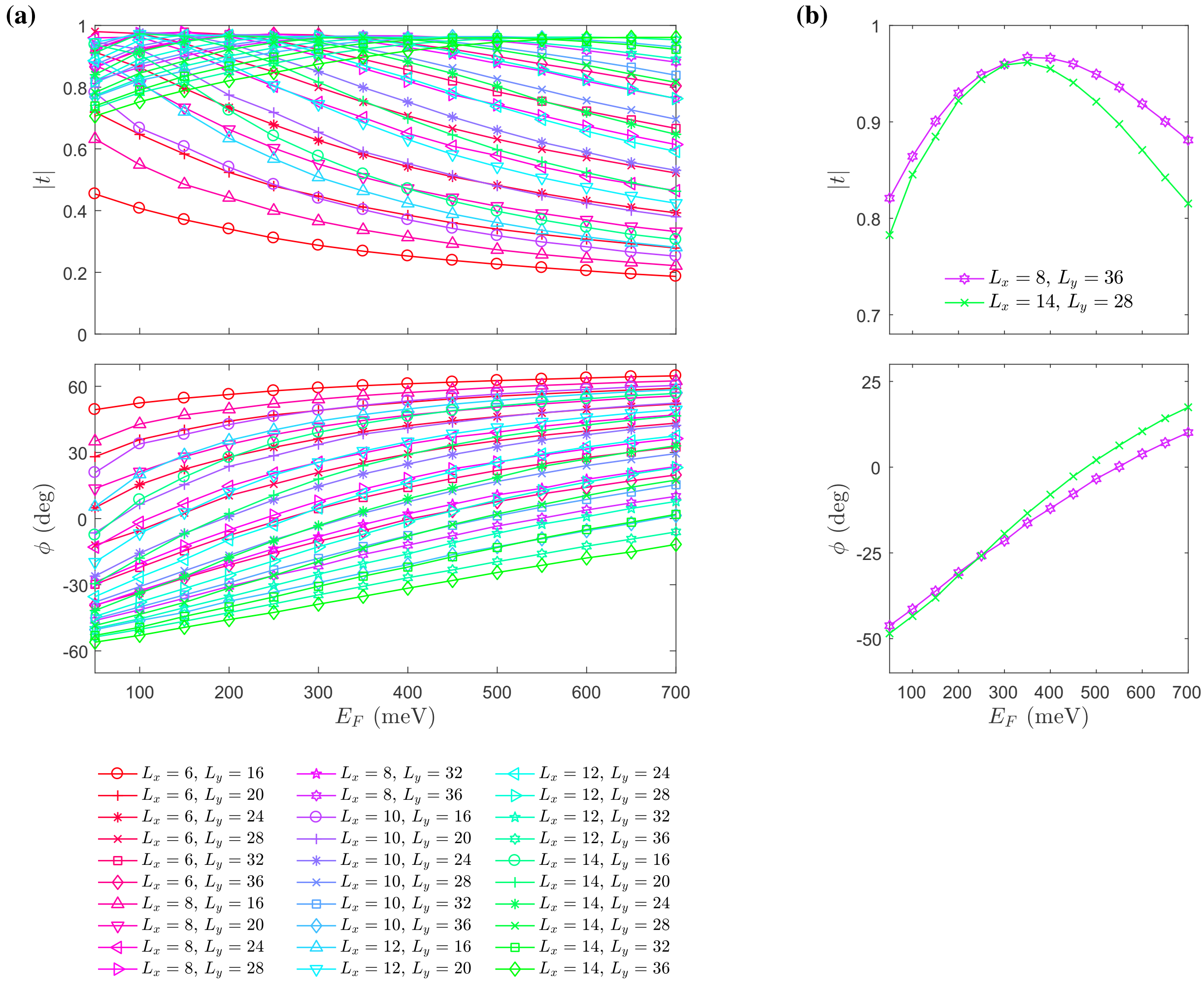

Figure S1. (a) Amplitude and phase response of the single-layer unit cell for different values of L_x and L_y at $f = 3$ THz as a function of graphene Fermi level, E_F. (b) Illustration of the amplitude and phase response for two selected cases with relatively higher amplitudes. For L_x and L_y, the values listed in the legends are in the unit of μm.

graphene patch length, L_g, is constant, which is set to 6 μm. Full-wave simulations do this examination for the Fermi level range of 50 meV to 700 meV, the results of which are arranged in Fig. S1. As it is clear from this figure, the curves whose colors are near to green (which correspond to larger values of L_x) have larger amplitudes for the transmission response. However, we also have a large phase change for different values of Fermi level. Based on these criteria (high transmission and large phase change), we select the first case, i.e., $(L_x, L_y) = (14, 28)$ μm. In Fig. S2(a), we plot the transmission responses of the unit cell for constant $L_x = 14$ μm but different values of L_y. It is evident from this figure that the best choice is $(L_x, L_y) = (14, 28)$ μm (the dotted curve) because not only the transmission amplitude is high and flat, but it also has a large and almost linear phase change versus E_F.

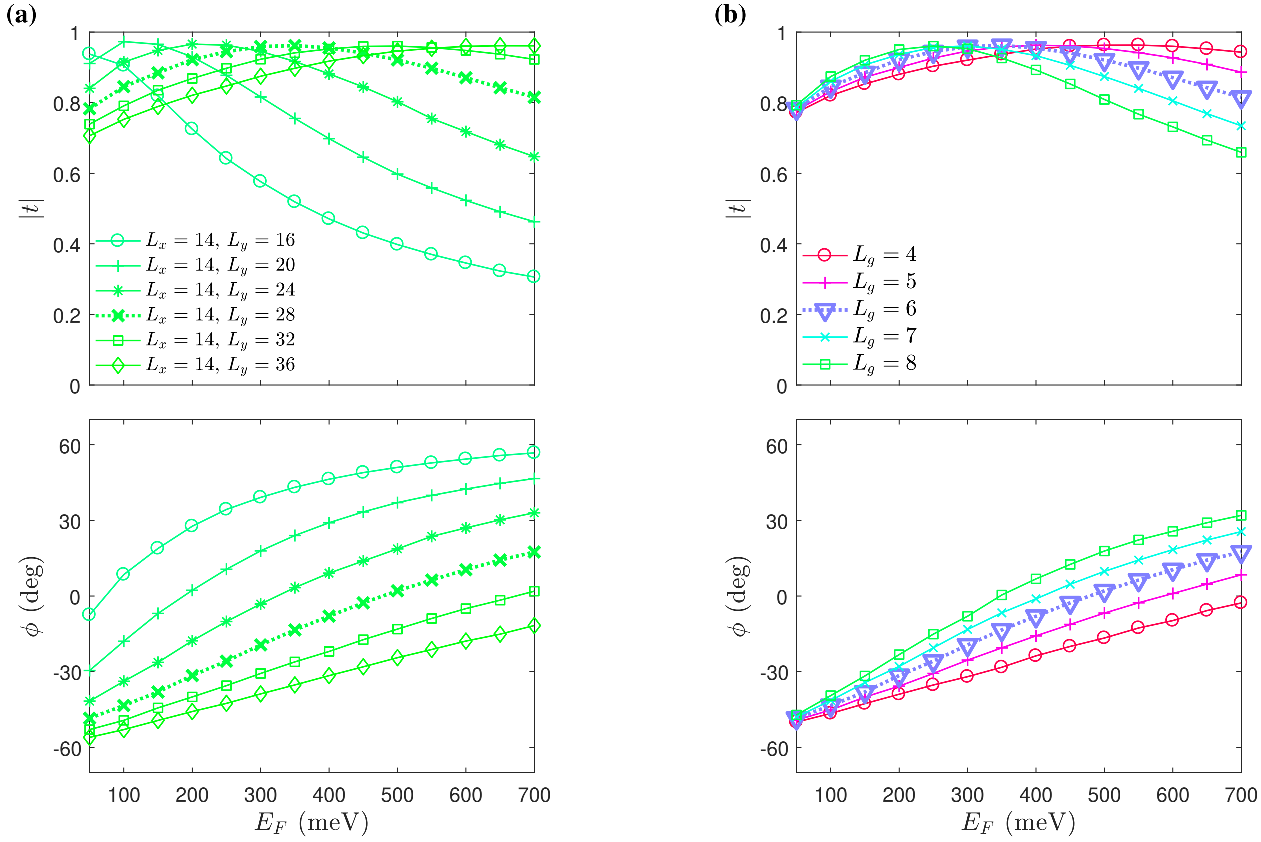

Figure S2. (a) Amplitude and phase response of the single-layer unit cell for different values of L_y at $f = 3$ THz versus graphene Fermi level, E_F, when $L_x = 14$ μm and $L_g = 6$ μm. (b) Amplitude and phase response of the single-layer unit cell for different values of L_g, in which the other geometric parameters are the same as those of the designed structure proposed previously in the manuscript: $P_x = 18$, $P_y = 30$, $L_x = 14$, $L_y = 28$ and $W_S = 2$, all in the unit of μm.

As the next step in design procedure, we study the graphene patch length, L_g, and investigate the transmission response of the proposed unit cell. As it is obvious in Fig. S2(b), there is a trade-off between the transmission phase change and the transmission amplitude, i.e., if we increase L_g, the transmission phase change increases (desired), but the amplitude drops for some values of Fermi level (undesired). We select the case in which $L_g = 6$ μm because the amplitude response is almost symmetric over the entire range of E_F, and the transmission phase change is acceptable.

1.2. The Unit Cell Frequency Response

According to Fig. 3(a) in the manuscript, the transmission phase alters when the Fermi level of graphene patches varies from 50 to 700 meV for the single-layer graphene metasurface, the schematic of which is illustrated in Fig. 2. As stated in the manuscript, the transmission phase shifts owing to the interactions between the graphene patch and H-shaped etched metal film. Here, we want to demonstrate the effect of graphene patch on the transmission coefficient versus frequency for the single-layer metasurface composing the proposed transmit-array. By embedding the graphene patches within the H-shaped etched metal film structures, the transmission response is modified. In fact, the interactions between the graphene and the etched metal film, which results in a shift of transparent window toward higher frequencies, can be controlled by the graphene Fermi level. The amplitude and phase of shifted transparent windows formed by the single-layer metasurface

with different Fermi level values are plotted in Fig. S3. Cascading four same layers of these transparent windows whose transmission coefficient are depicted in Fig. S3, we achieve 360° phase control although the transmission amplitude and the bandwidth decrease, see Fig. 5(b) in the manuscript.

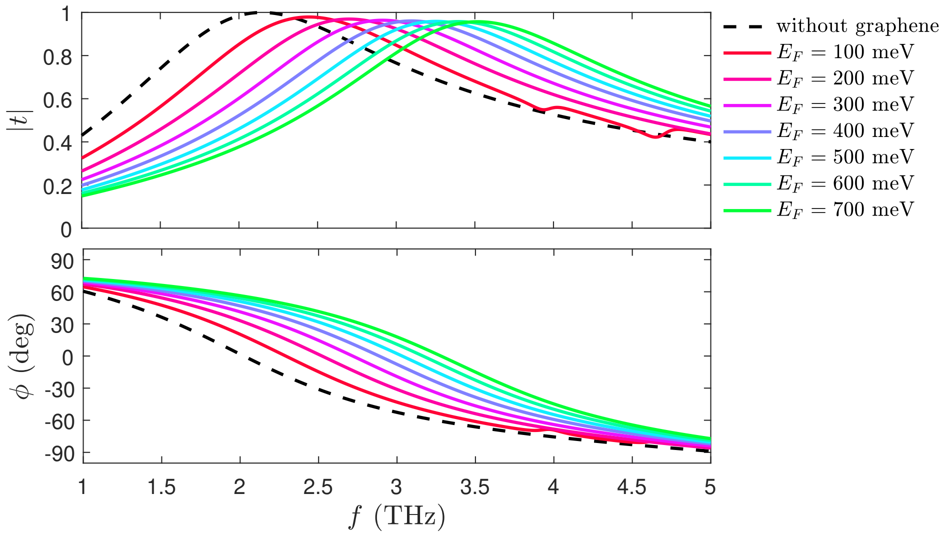

Figure S3. Amplitude and phase of the transmission coefficient of the single-layer metasurface whose uint cell is made up an H-shaped etched metal film together with a graphene patch for different Fermi level values.

2. Dispersion of the Target Waveguide

The artificial plasmonic metal structure constituted by a dielectric-coated metal sheet is our target structure, which supports SWs. The dispersion of such a structure is studied using the transverse resonance method (TRM) which makes use of a transmission line model of the waveguide transverse cross section [S1]. This analytical method provides a simple and direct solution to find the waveguide dispersion. The propagation constant, beta can be found using transverse resonance condition

$$Z^{\mathrm{down}} + Z^{\mathrm{up}} = 0 \tag{S1}$$

where Z^{up} and Z^{down} are the input impedances seen looking to the up- and downsides of an arbitrary reference plane. By employing this method, the dispersion of the waveguide schematically depicted in Fig. S4(a) can be found using the equivalent transverse circuit model shown in Fig. S4(b). Considering Eq. S1, the characteristic equation for the waveguide for TM modes is calculated as

$$jZ_1 \tan\left(k_{1z} t_{\mathrm{PM}}\right) + Z_2 = 0 \tag{S2}$$

where $k_{1z} = k_0 n_1 \sqrt{1-\left({}^{\beta}/_{k_0 n_1}\right)^2}$, $Z_1 = \left({}^{\eta_0}/_{n_1}\right)\sqrt{1-\left({}^{\beta}/_{k_0 n_1}\right)^2}$, and $Z_2 = -j\left({}^{\eta_0}/_{n_2}\right)\sqrt{\left({}^{\beta}/_{k_0 n_2}\right)^2-1}$ (k_0 and η_0 are wavenumber and wave impedance of vacuum). In Eq. S2, the first term corresponds to the short-circuited line impedance, Z^{down}, and the second term corresponds to the characteristic impedance of the infinite line, Z^{up}.

We plot the waveguide dispersion diagram in Fig. S5(a) in which the results are carried out based on both TRM and finite element method (FEM). Furthermore, as shown in Fig. S5(b), the normalized propagation constant is plotted as a

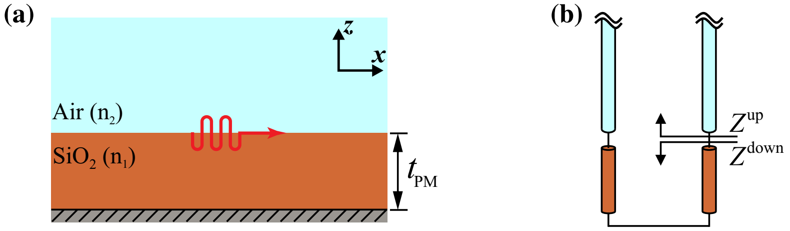

Figure S4. (a) Geometry of the target waveguide implemented by a grounded ultra-thin dielectric slab. The red arrow specifies the direction of the propagating SW mode. (b) Corresponding equivalent transverse circuit for SW modes calculation.

function of the dielectric thickness. It should be reminded that we consider the first propagating surface wave mode for this structure, whose cutoff frequency is equal to zero [S1].

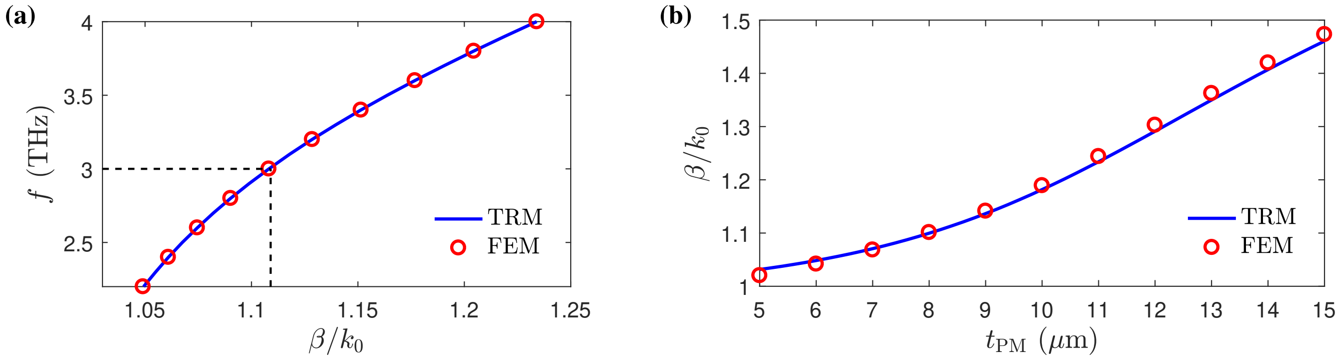

Figure S5. (a) Dispersion relation of TM_0 SW mode for the grounded 8.2-μm-thick dielectric slab. (b) Normalized propagation constant versus the dielectric thickness at $f = 3$ THz.

3. The Biasing Architecture for the Configuration

In this section, we explain the biasing architecture for the active tuning of graphene patches in the proposed configuration. The biasing mechanism is implemented by virtue of polysilicon pads, details and considerations of which are described in the following.

3.1. Considerations Associated With Polysilicon Pads

Using polysilicon pads positioned in a SiO_2 host is one of the most common methods for active tunning of graphene [S2, S3, S4]. By applying DC voltages to the polysilicon pads, one may control the Fermi levels electrostatically in each meta-atom. Being extremely thin and having similar permittivity to that of the SiO_2 substrate, polysilicon pads reliably can be neglected [S5]. However, Here, we take into account the polysilicon pads in our simulations, and demonstrate that considering and not considering these polysilicon DC gating pads have almost the same results.

Firstly, we examine the transmission amplitude and phase of the four-layer unit cell in which we have considered polysilicon pads in each layer. As plotted in Fig. S6, there are slight differences in amplitude and phase responses between

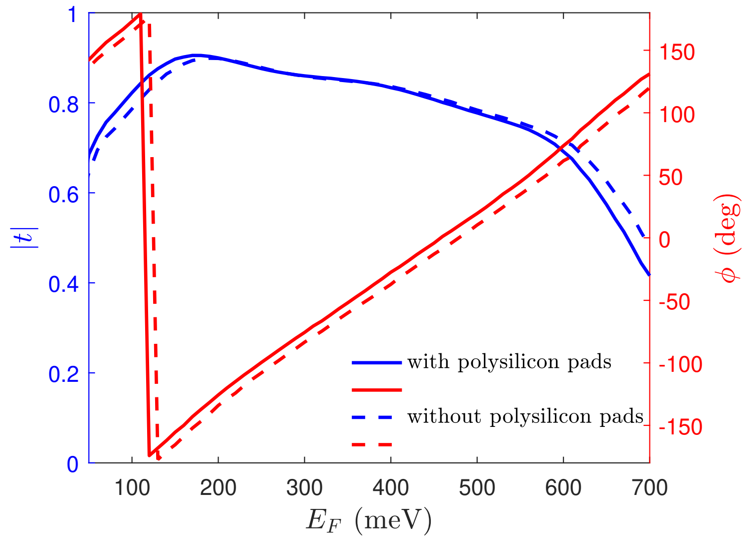

Figure S6. Transmission amplitude (left axis) and phase (right axis) carried out based on full-wave simulations versus Fermi level of graphene patches for the proposed transmitarray at $f = 3$ THz. The solid and dashed lines correspond to the cases in which we consider the polysilicon pads, and ignore them, respectively.

two cases, in one of which we consider polysilicon pads. These minimal deviations between these two cases tell us that ignoring polysilicon pads is logical and acceptable. We should add that the small shift between the phase responses for these two cases is not important in our design procedure (the absolute values of phase responses is not important), because the phase difference between unit cells determines the performance of our device. Thus, we expect that final results, i.e., coupling efficiency and directivity for both cases (with/without polysilicon pads) to be identical. Here, we will verify this expectation by full-wave simulation for the whole structure.

Now, we consider the polysilicon pads in the whole structure with optimized distance between meta-coupler and target waveguide ($h = 40\ \mu\mathrm{m}$), and obtain the total coupling efficiency and directivity. For this case, based on full-wave simulations, the values for efficiency and directivity are 46% and 10 dB, respectively, which are equal to the case where pads have been ignored. It is worth mentioning that we consider local mesh refinement for the polysilicon pads (owing to their ultra-thin thickness), which leads to time-consuming simulations.

3.2. Voltage Values for Polysilicon Gating DC Pads

For tuning the Fermi level of graphene patches in our proposed meta-coupler, we make use of the explained architecture by which the independent control of Fermi level values is possible as described in the manuscript. Herein, some details associated with the biasing architecture employed in our reconfigurable meta-coupler should be highlighted. We obtain the electrostatic field bias E_b by which the graphene patch Fermi level of each meta-atom can be adjusted as follows [S6, S5]:

$$n_s = \frac{2}{\pi\hbar^2 v_f^2}\int_0^\infty \epsilon\left[f_0\left(\epsilon - E_F\right) - f_0\left(\epsilon + E_F\right)\right] d\epsilon \tag{S3a}$$

$$E_b = \frac{e n_s}{\epsilon_0 \epsilon_r^{\mathrm{dc}}} \tag{S3b}$$

where n_s is the graphene carrier density, $\hbar$ is the Planck constant, $v_f \sim 10^6\ \mathrm{m/s}$ is the Fermi velocity, e is the electron charge and ϵ_r^{dc} is relative permittivity of the dielectric at DC. In this equation $f_0(\epsilon) = \left(1 + \exp\left(\left(\epsilon - E_F\right)/k_B T\right)\right)^{-1}$ is the Fermi-Dirac distribution where k_B and T denote Boltzmann constant and temperature, respectively. Therefore, the bias voltage can be calculated simply by

$$V_b = \frac{e n_s}{C_g} \tag{S4}$$

where $C_g = \epsilon_0 \epsilon_r^{\mathrm{dc}}/d_g$ is the gate capacitance of electrodes per unit area, and d_g is the electrodes distance. Concerning the biasing architecture, $d_g = h_p = 50\ \mathrm{nm}$, and for the $\mathrm{SiO_2}$ dielectric, $\epsilon_r^{\mathrm{dc}} = 3.9$. Considering Eqs. S3 and S4, we calculate electrostatic field and voltage biases for the five meta-atoms constituting the meta-coupler supercells. The values of field and voltage biases are tabulated in Table S1.

TABLE S1. REQUIRED ELECTRIC FIELD BIASES FOR STATES I AND II.

State I					
Meta-Atom	#1	#2	#3	#4	#5
E_F (meV)	626	486	331	189	70
E_b (V/nm)	0.93	0.56	0.26	0.09	0.02
V_b (V)	46.6	28.2	13.2	4.5	0.8

State II					
Meta-Atom	#1	#2	#3	#4	#5
E_F (meV)	70	189	331	486	626
E_b (V/nm)	0.03	0.09	0.26	0.56	0.93
V_b (V)	0.8	4.5	13.2	28.2	46.6

The 3D schematic of the biasing architecture of the proposed meta-coupler for both state I and sate II are represented by Fig. S7. The DC bias voltages are applied to polysilicon pads acting as electrodes. The micro-controller unit (MCU) in the biasing structure is responsible for controlling the values of voltage biases in such a way that the meta-coupler operates in state I or II.

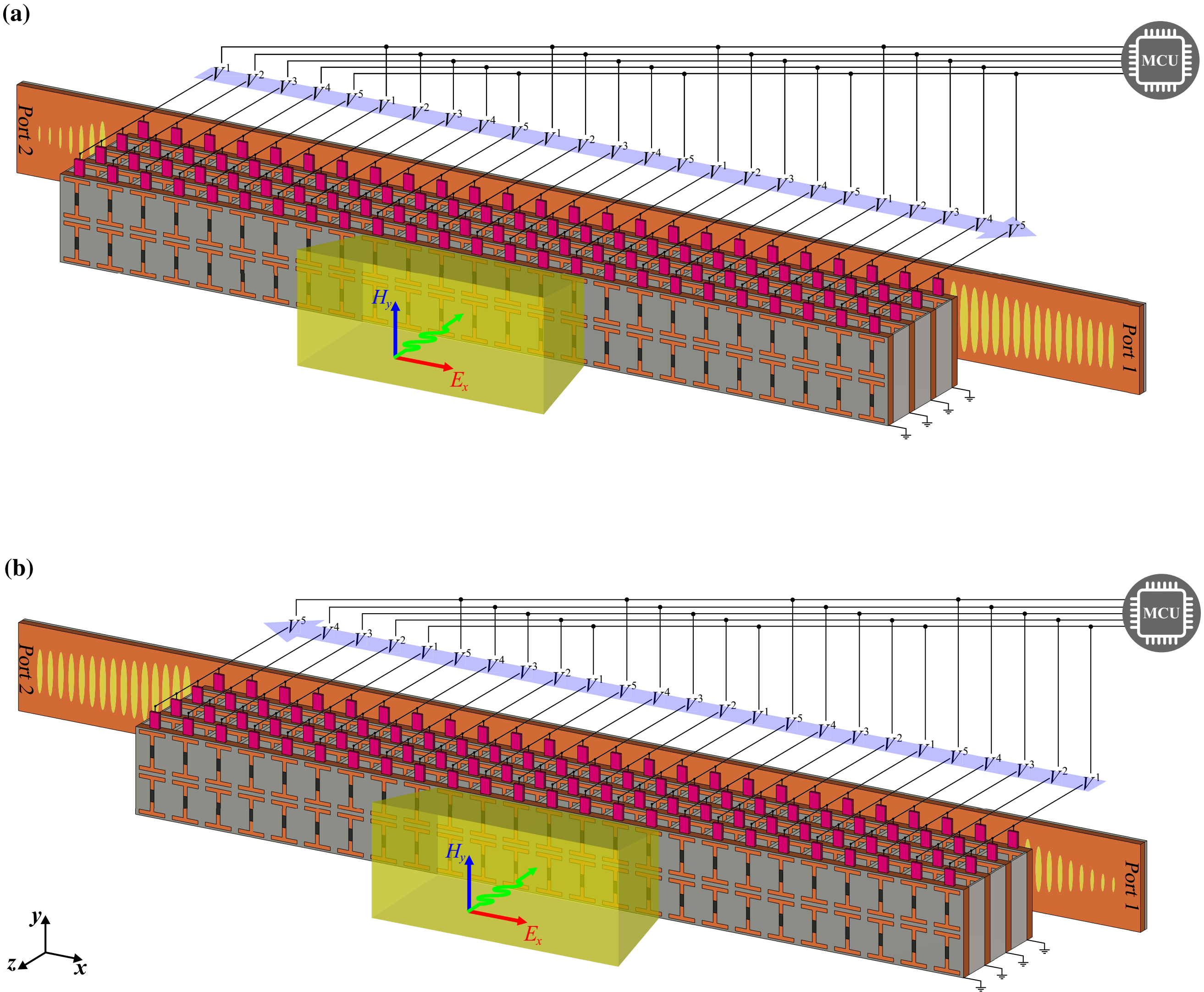

Figure S7. Biasing architecture of the graphene-based meta-coupler for (a) state I and (b) state II. In states I and II, the incident propagating wave in the opposite of z-direction is coupled to ports 1 and 2, respectively. The Fermi level values of graphene patches in meta-atoms are labeled below them in each supercell. Orders of the Fermi level values corresponding to states I and II are in reverse to each other (the blue arrows in (a) and (b) indicate these orders). By changing the order of the Fermi level values from left-to-right to right-to-left, the device can be switched from state I to state II. In these illustrations, the yellow regions depict illuminating x-polarized Gaussian beams.

References

[S1] D. M. Pozar, *Microwave engineering*. John Wiley & Sons, 2009.

[S2] P.-Y. Chen, C. Argyropoulos, M. Farhat, and J. S. Gomez-Diaz, "Flatland plasmonics and nanophotonics based on graphene and beyond," *Nanophotonics*, vol. 6, no. 6, pp. 1239–1262, 2017.

[S3] J.-S. Gómez-Díaz and J. Perruisseau-Carrier, "Graphene-based plasmonic switches at near infrared frequencies," *Optics express*, vol. 21, no. 13, pp. 15 490–15 504, 2013.

[S4] J. Gomez-Díaz, C. Moldovan, S. Capdevila, J. Romeu, L. Bernard, A. Magrez, A. Ionescu, and J. Perruisseau-Carrier, "Self-biased reconfigurable graphene stacks for terahertz plasmonics," *Nature communications*, vol. 6, p. 6334, 2015.

[S5] M. Esquius-Morote, J. S. Gómez-Díaz, and J. Perruisseau-Carrier, "Sinusoidally modulated graphene leaky-wave antenna for electronic beamscanning at thz," *IEEE Transactions on Terahertz Science and Technology*, vol. 4, no. 1, pp. 116–122, 2014.

[S6] G. W. Hanson, "Dyadic green's functions and guided surface waves for a surface conductivity model of graphene," *Journal of Applied Physics*, vol. 103, no. 6, p. 064302, 2008.
